# Optimized method development and validation for determining donepezil in rat plasma: A liquid-liquid extraction, LC-MS/MS, and design of experiments approach

**DOI:** 10.1371/journal.pone.0309802

**Published:** 2024-09-06

**Authors:** Ye Bin Shin, Jin Hwan Kim, Min Kyeong Kwon, Jin Hyuk Myung, Dong Geon Lee, Sung Giu Jin, Myung Joo Kang, Yong Seok Choi

**Affiliations:** 1 College of Pharmacy, Dankook University, Cheonan, Chungnam, South Korea; 2 Department of Pharmaceutical Engineering, Dankook University, Cheonan, Chungnam, South Korea; University of Glasgow School of Medicine: University of Glasgow School of Medicine Dentistry and Nursing, UNITED KINGDOM OF GREAT BRITAIN AND NORTHERN IRELAND

## Abstract

Donepezil (DPZ), a piperidine-based reversible cholinesterase inhibitor, finds extensive use in treating Alzheimer’s disease (AD). Originally designed as an oral formulation, DPZ encounters drawbacks such as a brief duration of action and reduced treatment effectiveness in elderly patients with memory impairment or difficulty swallowing medications. To address these issues and improve patient compliance, researchers are actively exploring alternative DPZ formulations. Consequently, reliable methods are necessary to quantitate DPZ in biological samples for *in vivo* assessment. Therefore, we propose an efficient, sensitive, wide-dynamic, and cost-effective method for quantitating DPZ in rat plasma. Our method employs liquid-liquid extraction (LLE) followed by liquid chromatography and tandem mass spectrometry, enabling *in vivo* evaluation of novel DPZ formulations. Notably, our method requires only 20 μL of rat plasma and employs icopezil as the internal standard—a cost-effective compound with chemical similarity to DPZ. We meticulously optimized LLE conditions, taking into account factor interactions through design of experiments (DOE). Our rapid and straightforward extraction and purification involved using 500 μL of pure methyl *tert*-butyl ether to extract DPZ from the sample within five minutes. The dynamic range of the method extends from 0.5 ng/mL to 1,000 ng/mL, demonstrating excellent sensitivity and suitability for pharmacokinetic studies across diverse DPZ formulations. Following the FDA guidelines, we rigorously validated the developed method, evaluating selectivity, linearity (with a coefficient of determination ≥0.9999), accuracy (ranging from 96.0% to 109.6%), precision (≤13.9%), matrix effect (92.2% to 103.8%), recovery (98.5% to 106.8%), the lower limit of quantitation (0.5 ng/mL), and stability. Finally, we effectively employed the validated method for the long-term pharmacokinetic assessment of a DPZ formulation. We expect that this approach will make a substantial contribution to the advancement of new DPZ formulations, ultimately benefiting individuals afflicted by AD.

## Introduction

Alzheimer’s disease (AD) is a progressive neurodegenerative disease, and it was the seventh-leading cause of death in the United States (US) in 2021 [1]. It is estimated that more than 6.9 million Americans older than age 65 (about 73% of whom are older than age 75) live with AD. Due to the growing incidence of AD and related dementias, the estimated cost of caring for people with these diseases in the US will increase from $360 billion in 2023 to just under $1 trillion in 2050 [1]. Unfortunately, it is impossible to definitively diagnose AD during a patient’s lifetime. The only way to confirm AD is through postmortem observation of the neuritic plaques and neurofibrillary tangles in the patient’s brain tissue [2]. As a result, there have been tremendous efforts to discover AD-diagnostic biomarkers from various sources, such as blood, cerebrospinal fluid (CSF), and even MRI images; however, no marker has been universally accepted by the medical community [3–8].

**Fig 1:**
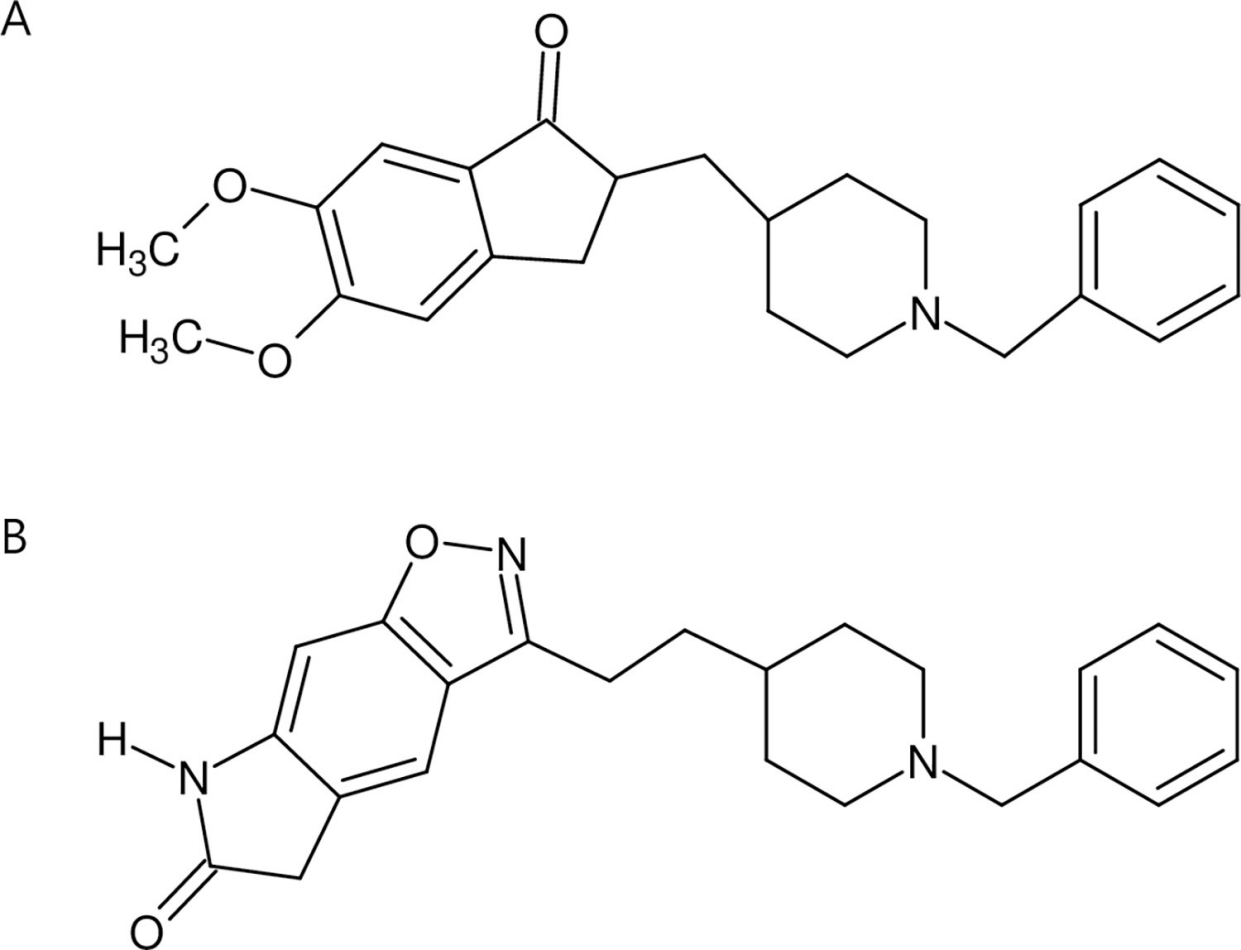
Chemical structures of donepezil (A) and icopezil (B).

Even in the treatment of AD based on medications, options are limited to three cholinesterase inhibitors (ChEIs), namely, donepezil, galantamine, and rivastigmine, and a NMDA receptor antagonist (memantine) [9,10]. Among these four drugs, donepezil has demonstrated the largest market share [11]. Donepezil (DPZ, Fig 1), a piperidine-based reversible ChEI, is widely used in the treatment of AD. It plays a crucial role in alleviating the characteristic symptoms of AD, such as cognitive impairment and memory loss, which result from the reduction of the neurotransmitter acetylcholine in the brain [9,10]. Initially developed as an oral formulation, DPZ faces the disadvantage of short duration of action and may lead to decreased treatment efficacy in elderly patients with amnesia or difficulty swallowing tablets or capsules [9]. To address these challenges and enhance patient compliance, a transdermal formulation of DPZ was developed [12]. Additionally, long-acting injectable formulations are actively under study [13]. Therefore, efficient, sensitive, and wide-dynamic methods to determine DPZ in biological samples are essential for the *in vivo* evaluation of its new formulations.

Recently, most methods for quantitating DPZ in biological samples utilize liquid chromatography and tandem mass spectrometry (LC-MS/MS), particularly multiple reaction monitoring (MRM) due to its high selectivity and sensitivity [14–20]. However, when using LC-MS/MS, the sample preparation step must be meticulously designed to prevent signal suppression among co-existing compounds during electrospray ionization (ESI) [21,22]. For DPZ studies based on LC-MS/MS, two common approaches are protein precipitation and liquid-liquid extraction (LLE) [14,15,17–20]. While protein precipitation effectively removes proteins from the sample, it may not adequately address the suppression effect on DPZ, a nonpolar compound with a logP value of 4.3, due to its inability to eliminate other nonpolar compounds, such as phospholipids [23,24]. Consequently, protein precipitation could adversely impact the quantitative performance of the method. In contrast, LLE proves to be a superior sample preparation method compared to protein precipitation when evaluating sensitivity and matrix effects for the determination of DPZ in plasma [25]. This superiority arises from LLE’s ability to remove nonpolar compounds from the sample, which can be adjusted by varying the extraction solvent [14,15]. Although modern analytical methods for DPZ have been reported, unfortunately, none of these methods have simultaneously achieved the three essential qualities: high efficiency requiring less than 50 μL of plasma, high sensitivity surpassing the lower limit of quantitation (LLOQ) of 1 ng/mL, and a wide dynamic range spanning at least three orders of magnitude. Since traditional approaches, which primarily focus on optimizing experimental factors through trial and error, likely contributed to this ‘sitting duck’ situation, it is rational to shift toward optimizing factor interactions using chemometric multivariate techniques [26–28]. The design of experiments (DOE) has been widely employed for chemometric multivariate analysis to optimize various chemical research processes efficiently with a small number of experiments [26–28].

Thus, in this study, we propose an efficient, sensitive, wide-dynamic, and cost-effective method for determining DPZ in rat plasma. Our approach includes an extraction and purification (E/P) step using LLE, which we comprehensively optimized using the DOE for considering factor interactions. Subsequently, we employed LC-MS/MS, specifically MRM. We thoroughly validated our developed method and compared it with existing approaches. Finally, we successfully confirmed the applicability of the validated method in an actual pharmacokinetic study on DPZ formulations.

## Materials and methods

### Chemicals and reagents

DPZ (≥99%) and icopezil (the internal standard (IS), Fig 1, ≥99%) were procured from Sigma-Aldrich (St. Louis, MO, USA) and Biorbyt (Durham, NC, USA), respectively. Formic acid (LC-MS grade) was sourced from Sigma-Aldrich, and water, acetonitrile, and methyl *tert-*butyl ether (MTBE) were obtained as HPLC grade from J. T. Baker (Phillipsburg, NJ, USA).

### Standard solutions preparation

Stock solutions of DPZ and the IS were prepared by dissolving 1 mg of each in methanol to a concentration of 1 mg/mL, and they were stored at -80°C, until use. The DPZ stock solution was diluted with methanol to prepare working solutions (100 and 1 ng/mL). Additionally, the extraction solvent was prepared by diluting the IS stock solution with MTBE to 200 ng/mL. All working solutions, including the extraction solvent, were stored at -20°C, until use.

### Sample preparation

An aliquot (20 μL) of rat plasma was mixed with 500 μL of the extraction solvent, which contained the IS for five minutes. After centrifuging the mixture at 1,240 ×g for five minutes, the entire upper layer (the extraction solvent layer) was transferred to a new 1.5 mL-microcentrifuge tube. The solution was then evaporated to dryness at room temperature under a stream of nitrogen. The dried residue was re-dissolved in 100 μL of acetonitrile. The reconstituted solution underwent centrifugation at 1,240 ×g for five minutes, and a portion of the supernatant was analyzed by LC-MS/MS. A matrix-matched standard (MMS) and a standard-spiked sample (SSS) were prepared by adding an appropriate volume of a DPZ working solution to the prepared blank rat plasma extract and to blank rat plasma prior to sample preparation steps, respectively. For example, MMSs were employed as calibrators (0.5, 1.5, 5, 50, 250, 500, 1,000 ng/mL), and SSSs were used as QC samples (0.5, 1.5, 500, 1,000 ng/mL). In all solutions analyzed by LC-MS/MS, the concentration of IS was maintained at 100 ng/mL.

### Liquid chromatography and tandem mass spectrometry (LC–MS/MS)

Basically, a Shimadzu Nexera UPLC system (Tokyo, Japan) and a Shimadzu LCMS 8050 triple quadrupole mass spectrometer were interfaced through ESI in positive ion mode. For chromatographic separation, a Phenomenex Luna Omega Polar C18 column (2.1×100 mm, 3 μm, Torrance, CA, USA) was employed under gradient mobile phase conditions consisting of 0.1% (v/v) formic acid in water (A) and 0.1% (v/v) formic acid in acetonitrile (B), with a flow rate of 0.25 mL/min. During the initial 1.50 minutes, only 100% (v/v) of mobile phase A was maintained, followed by an increase to 70% (v/v) of mobile phase B at 1.60 minutes. This mobile phase condition was sustained until 5.00 minutes, after which the portion of mobile phase B was reduced back to 0% (v/v) at 5.10 minutes and maintained until the end of the separation (8.00 minutes). The autosampler and column oven were maintained at 4°C and 40°C, respectively.

For ESI, the system settings were as follows: DL temperature of 250°C, interface temperature of 380°C, heating block temperature of 400°C, dry gas flow rate of 10 L/min, heating gas flow rate of 10 L/min, and nebulizing gas flow rate of 3 L/min. Among MS/MS scan modes, MRM was selected for selective and sensitive determination of DPZ. A total of three MRM transitions per DPZ or the IS were monitored. For DPZ, 380.2 *m/z* (precursor ion) / 91.2 *m/z* (product ion) / -39 V (collision energy) was set for the screening transition, and 380.2 *m/z* / 65.2 *m/z* / -86 V and 380.2 *m/z* / 243.3 *m/z* / -26 V were used as confirmatory transitions. In the case of the IS, 376.3 *m/z* / 91.2 *m/z* / -31 V, 376.3 *m/z* / 284.3 *m/z* / -23 V, and 376.3 m/z *m/z* / 212.3 *m/z* / -23 V were employed as its screening transition, the first confirmatory transition, and the second confirmatory transition, respectively (Table 1). All LC-MS/MS data were processed using Shimadzu Lab Solutions software (version 5.93). For quantitation, the pre-requirements were checked: all three transition peaks must have the same retention time, the signal-to-noise ratio (S/N) of the screening transition peak must be higher than 10, and the S/N value of all confirmation transition peaks must be higher than 3. After meeting all the pre-requirements, the screening transition peak area ratio of DPZ to the IS was used for quantitation.

**Table 1:** Properties of donepezil and icopezil.

Compound	Exact mass (Da)	Retention time (minutes)	MRM transitions		
			Precursor ion (*m/z*)	^a^Product ion (*m/z*)	^b^CE (V)
Donepezil	379.21	3.2	380.20	91.20	-39
				65.20	-86
				243.30	-26
Icopezil	375.19	3.2	376.25	91.15	-31
				284.25	-23
				212.25	-23

^a^ The product ion of a screening transition; the product ion of a confirmatory transition.

^b^ Collision energy; the CE of a screening transition; the CE of a confirmatory transition.

### Method validation

The developed method was validated in accordance with the FDA guidelines, specifically the ‘M10 Bioanalytical Method Validation and Study Sample Analysis’ [29]. The validation covered several parameters, including selectivity, linearity, accuracy, precision, matrix effect (ME), recovery (R), sensitivity (LLOQ), and stability.

#### Selectivity

Selectivity was assessed by comparing results from blank rat plasma with those from the LLOQ QC sample (0.5 ng/mL, n = 6). Blank rat plasma results should exhibit no interference at the retention times of DPZ and the IS, with less than 20% of the response from DPZ at LLOQ and less than 5% of that from the IS.

#### Linearity

Linearity was evaluated using MMSs of seven concentrations ranging from 0.5 to 1,000 ng/mL (n = 6). Calibration curves were constructed using simple linear regression, and the coefficient of determination (r^2^) was calculated for each curve. Additionally, the accuracy and precision between the nominal and back-calculated concentrations of each calibrator were compared. Accuracy is expressed as the percentage of mean back-calculated concentration to its nominal concentration, while precision is represented by the coefficient of variation (CV, %) of accuracy. The accuracy at LLOQ should be within 80–120%, and within 85–115% at all other concentrations.

#### Accuracy and precision

Intra-day accuracy and precision were determined by analyzing six replicates of QC samples within a single day. Inter-day accuracy and precision were evaluated over three consecutive days, with six replicates of QC samples analyzed daily. The accuracy at each concentration level should be within ±15% of the nominal concentration, and within ±20% at the LLOQ. The precision of the measured concentrations at each level should not exceed 15%, except at the LLOQ where it should not exceed 20%.

#### Matrix effect (ME) and recovery (R)

The ME was assessed by comparing results from MMSs at concentrations of LLOQ QC, low QC (LQC, 1.5 ng/mL), medium QC (MQC, 500 ng/mL), and high QC (HQC, 1,000 ng/mL) samples with those from the corresponding standard solutions (n = 6). The ratio of an MMS result to the mean standard solution result should be within 85–115% and the precision (%CV) should not be greater than 15%. R was determined by comparing results from QC samples with those from the conjugated MMSs at concentrations of LLOQ QC, LQC, MQC, HQC samples (n = 6). The ratio of an SSS result to the mean MMS result is not necessary to be 100%, but there should be consistency.

#### Sensitivity

The lowest concentration meeting criteria of accuracy and precision within the linear dynamic range was confirmed as the LLOQ. Additionally, the S/N of its screening transition peak and those of its confirmatory transition peaks should be higher than 10 and 3, respectively.

#### Stability

Stability evaluation was performed at DPZ concentrations of LQC and HQC samples under the following conditions (n = 6): Freeze-thaw stability in plasma was assessed after three cycles at -80°C. Benchtop stability in plasma was evaluated after four hours at room temperature. Long-term stability in plasma was assessed after storage for four weeks at -80°C. The stability of the extracted QC samples was investigated by re-analyzing them in an autosampler set at 4°C, 24 hours after the initial injection. Lastly, the stability of working solutions was evaluated following a week of storage at -20°C. The mean concentration measured at each level and under each condition should fall within 85–115% of the nominal concentration.

### Application to a pharmacokinetic study in rats

The validated method was employed to determine the plasma concentration-time profile of DPZ, following intramuscular (IM) injection of drug suspension. The drug suspension was prepared by suspending drug powder (hydrochloride salt of DPZ) in normal saline (100 mg/mL as DPZ). The animal study was carried out after the approval of the Institutional Animal Care and Use Committee (IACUC) of Dankook University (DKU-23-033, Cheonan, South Korea). Nine-week-old male Sprague-Dawley rats (300±20 g) acquired from Samtako Bio Korea (Gyeonggi-do, South Korea) were kept under controlled environmental conditions (23±1°C, 12 h day/ 12 h night) with free access to standard food and water. After three days of acclimatization, drug suspension was injected into the IM regions of the gastrocnemius at a dose of 24.5 mg/kg as DPZ. At predetermined times, rat blood samples (approximately 0.7 mL) were collected from the jugular vein using a heparinized 26-gauge needle-equipped syringe. The collected blood was centrifuged at 13,000 rpm for 10 minutes, and the resulting plasma was stored in a deep freezer at -80°C until its preparation and analysis using the validated method. Pharmacokinetic parameters of DPZ, such as the maximum drug concentration in plasma (C_max_), time to reach C_max_ (T_max_), area under the curve for drug concentration in plasma-time (AUC), and elimination half-life (T_1/2_), were calculated from the pharmacokinetic profile using Pharsight WinNonlin program (version 5.2, Mountain View, CA, USA).

## Results and discussion

### LC-MS/MS method

In this study, we chose icopezil as the IS due to its cost-effectiveness and its similar chemical structure and molecular weight to DPZ (Fig 1). We expected that icopezil would exhibit similar extraction characteristics and retention time as DPZ (Table 1). The IS is widely used in LC-MS/MS analyses of biological samples to correct for variability introduced by experimental steps such as E/P, LC, and MS/MS. Among the sources of variability, ionization suppression or enhancement of the analyte by co-eluting compounds at the LC-MS interface can pose a significant challenge. Therefore, it is crucial to select an IS with a retention time similar to that of the analyte to correct for ionization-related variability of the analyte. If the retention times of the analyte and the IS are similar, their ionization efficiencies may be similarly influenced by compounds that elute closely [30]. Other studies have used deuterated forms of DPZs, such as DPZ-d4 and DPZ-d7, as well as substances like loratadine and lansoprazole as ISs [10,17,19,20]. However, deuterated DPZs are expensive and less economical, and loratadine and lansoprazole have substantially different chemical structures from DPZ, potentially compromising accuracy. Therefore, our study aimed to improve both the economic efficiency and accuracy by selecting icopezil as the IS.

For MRM in positive ion mode, we selected [M+H]^+^ ions of DPZ and the IS with *m/z* values of 380.2 and 376.3, respectively, as precursor ions. To determine the product ions, we conducted product ion scans (PIS) for each precursor ion. From each PIS spectrum, we selected the most intense fragment ion for the screening transition (91.2 *m/z* for DPZ and 91.2 *m/z* for the IS). Additionally, we employed the second and third most intense fragment ions for the first and second confirmatory transitions (65.2 *m/z* and 243.3 *m/z* for DPZ, and 284.3 *m/z* and *212*.*3 m/z* for IS), respectively (Table 1). The screening transitions were used for quantitation purposes, while the confirmatory transitions were used for identity confirmation.

**Fig 2:**
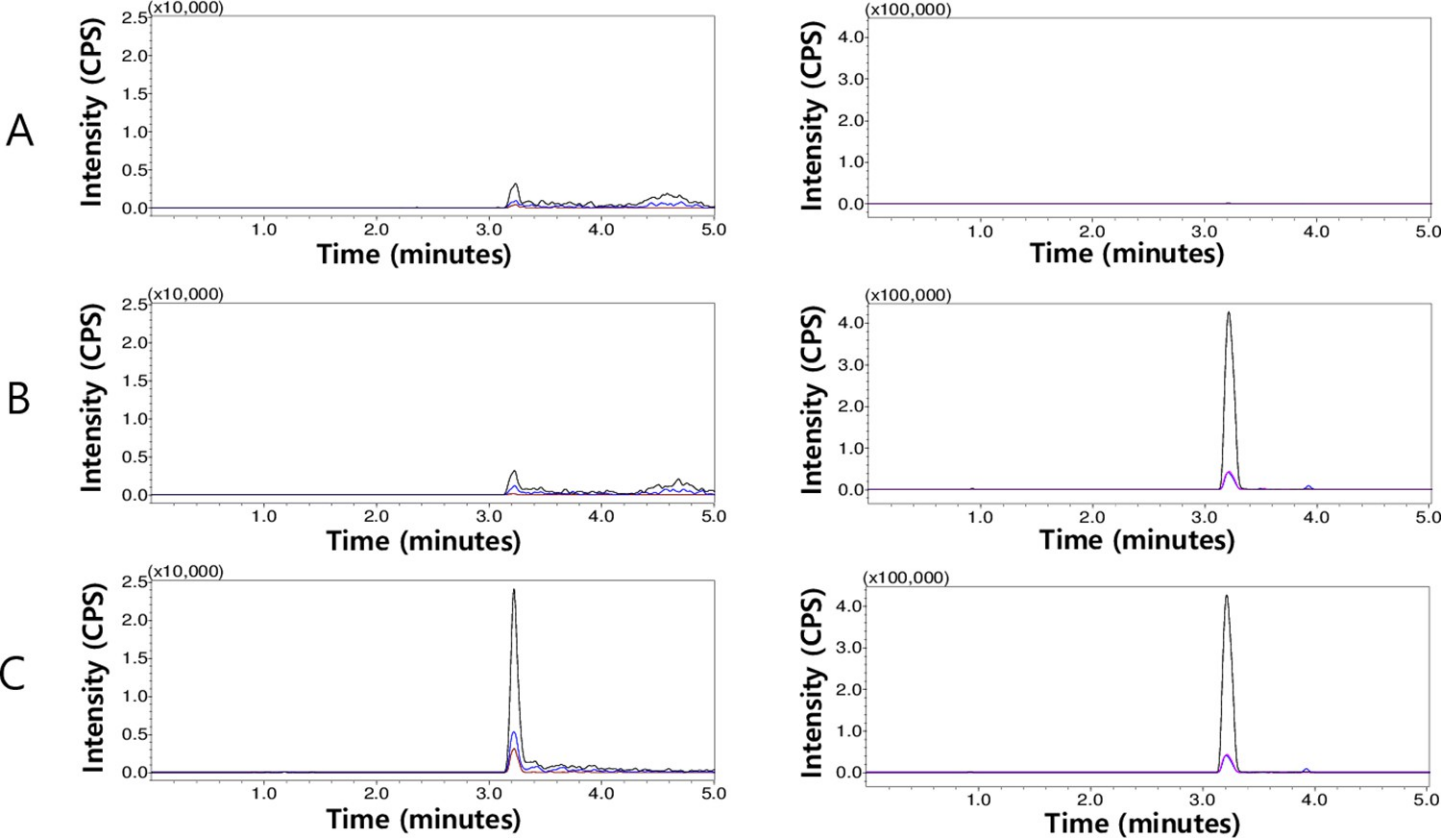
Multiple reaction monitoring chromatograms of donepezil (DPZ, left panels) and icopezil (internal standard, IS, right panels) in rat plasma: Double blank plasma (A), blank plasma with 100 ng/mL of the IS (B), and plasma with 0.5 ng/mL of DPZ and 100 ng/mL of the IS (C).

To optimize peak shape and sensitivity, we evaluated several types of C18 columns (Gemini NX-C18, Luna Omega Polar C18, and Kinetex PS C18, all from Phenomenex), along with organic modifiers (acetonitrile and methanol) and acid modifiers (formic acid and acetic acid). Among various combinations, the use of a Luna Omega Polar C18 column, acetonitrile, and formic acid significantly reduced peak tailing and resulted in the most distinct improvement in peak shape and sensitivity (S1 and S2 Figs in S1 File). This improved chromatographic result may be attributed to several factors. First, the low pH (approximately 3) of the mobile phase favors the existence of DPZ as a cation, specifically mono-protonated at its tertiary amine (pKa of around 9) [31]. Also, the presence of a halogen atom on the Luna Omega Polar C18 particle surface may lead to additional attraction between the cations and the lone pair electrons associated with the halogen atoms [32]. Regarding the IS, a similar explanation may apply due to its comparable chemical structure with DPZ (Fig 1). However, since precise pKa information for the IS is unavailable, this explanation remains less certain. Additionally, to prevent potential system contamination and matrix component carryover, we employed gradient mobile phase conditions rather than isocratic elution. The examples of resulting chromatograms can be found in Fig 2, and they also demonstrate the successful achievement of our initial goal to have similar retention times between DPZ and the IS (Table 1).

### Sample preparation

To achieve efficient E/P of DPZ from rat plasma, we utilized LLE in our present method. To efficiently optimize the LLE conditions under the consideration of factor interactions with minimal experiments, we conducted a DOE study. Initially, we employed a fractional factorial design (FFD) to identify essential factors and their interactions. Subsequently, we thoroughly optimized these factors, taking into account their interactions, through response surface methodology (RSM). As an initial reference, we consulted the LLE conditions from our prior method, which was employed for the determination of rotigotine (RTG) in rat plasma [22]. These conditions encompassed the sampling volume, extraction solvent, volume of the extraction solvent, and extraction temperature. Notably, both DPZ and RTG share comparable logP values (4.3 and 4.9, respectively), signifying their similar nonpolar characteristics [23,33]. Regarding the extraction solvent, apart from MTBE (previously employed for RTG extraction), we also assessed ethyl acetate. Ethyl acetate possesses a higher polarity index (4.4), rendering it more polar than MTBE (with a polarity index of 2.5) [34]. We conducted this evaluation because DPZ exhibits slightly higher polarity than RTG [23,33]. Additionally, although pH is a critical factor in LLE extraction, adjusting the sample plasma’s pH with an acid/base modifier was dismissed during the design phase because the method uses only a small volume (20 μL) of plasma.

**Fig 3:**
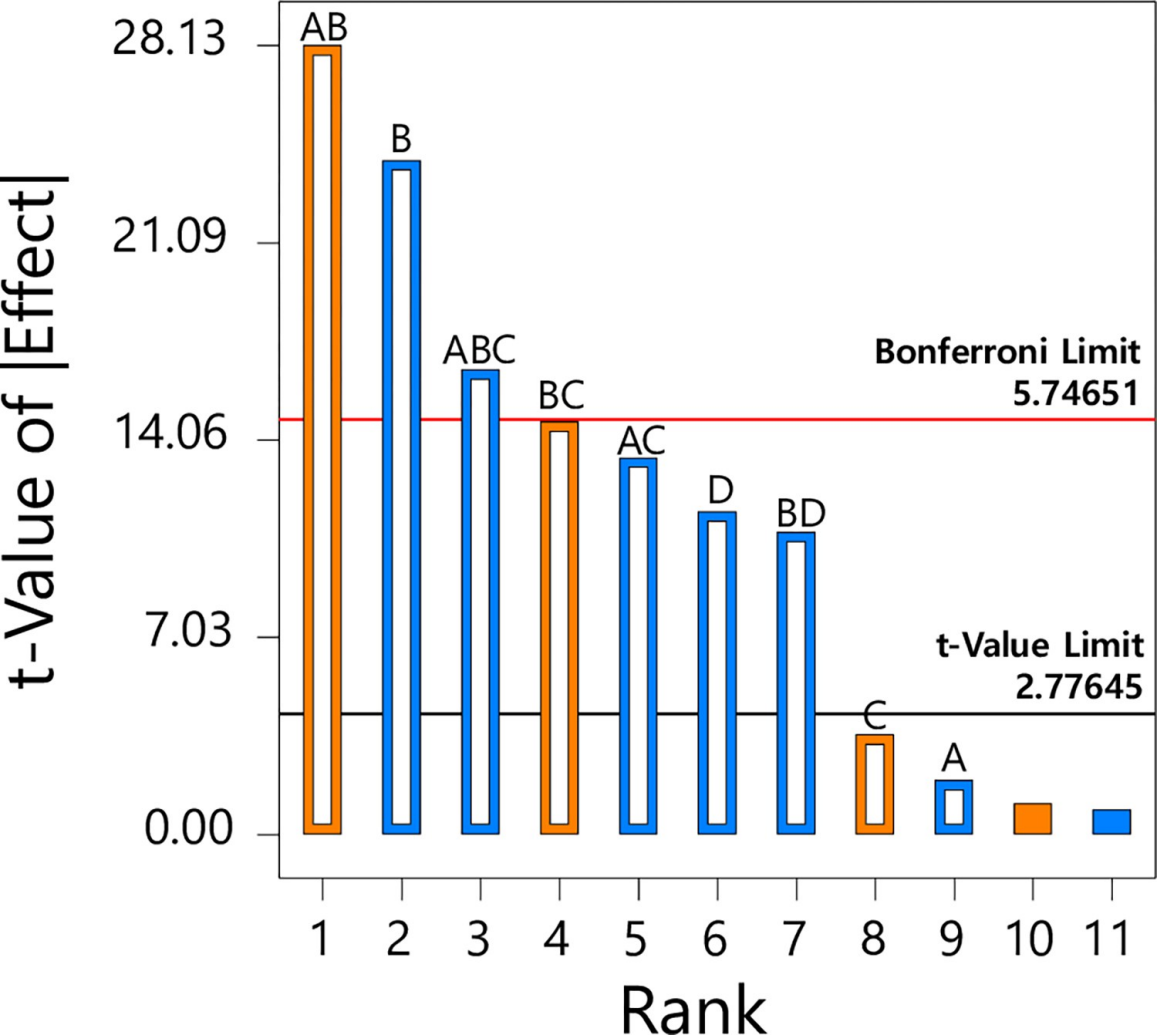
Pareto chart of the effects for extraction efficiencies of donepezil: Volumetric ratio of methyl *tert*-butyl ether to ethyl acetate in the extraction solvent (%, A), volume of the extraction solvent (μL, B), extraction duration (minutes, C), and extraction temperature (°C, D).

In the FFD, we assigned codes (A, B, C, and D) to each probably-significant factor influencing the extraction efficiency (expressed as R) of DPZ. Specifically, the code assignments were: A for the volumetric ratio of MTBE to ethyl acetate in the extraction solvent (ranging from 0% to 100% v/v); B for the volume of the extraction solvent (ranging from 100 μL to 900 μL); C for the extraction duration (5 to 45 minutes); and D for the extraction temperature (ranging from 0°C to 40°C). We configured these factors at both their lowest and highest levels, leading to 12 distinct experimental conditions (S1 Table in S1 File). Thus, a total of 12 experiments were conducted, and we identified critical factors and interactions that significantly influenced the extraction efficiency by analyzing the experimental conditions and their corresponding R values. Fig 3 illustrates that the factor and interactions AB, B, and ABC surpassed the Bonferroni limit, signifying crucial factors and interactions impacting the extraction efficiency. Remarkably, out of the three critical factors and interactions, two-thirds were factor interactions, underscoring the significance of optimizing these interactions in alignment with our primary research objective. Therefore, we incorporated the volumetric ratio of MTBE to ethyl acetate in the extraction solvent, the volume of the extraction solvent, and the extraction duration as key factors for optimization using RSM, taking their interactions into account.

**Fig 4:**
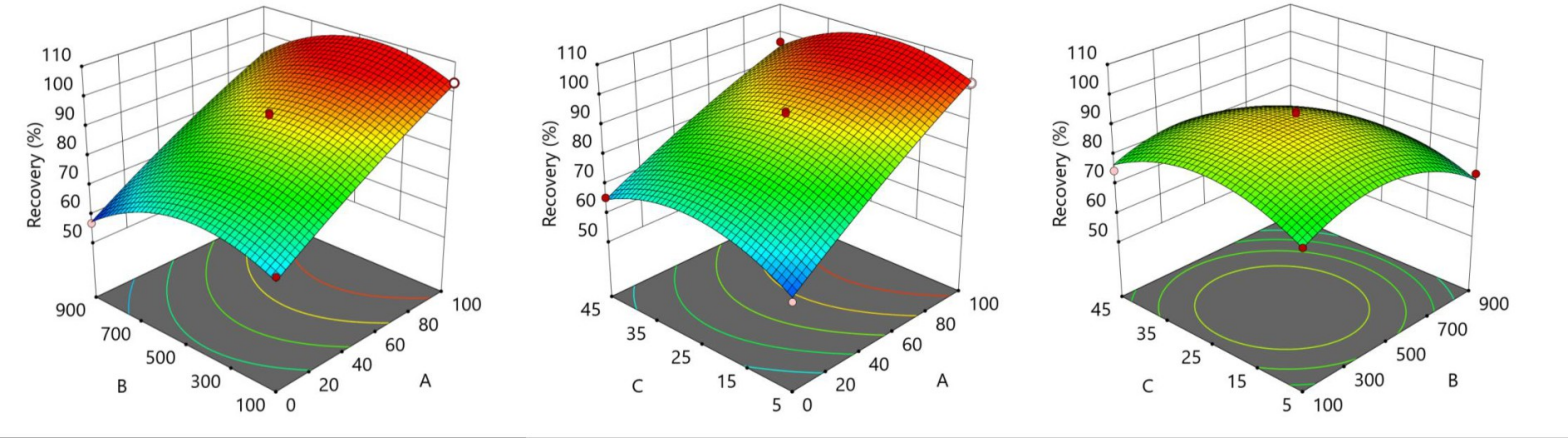
Response surface plots for effects of volumetric ratio of methyl *tert*-butyl ether to ethyl acetate in the extraction solvent (%, A), volume of the extraction solvent (μL, B), and extraction duration (minutes, C) on extraction efficiency.

To optimize using RSM, we employed a Box-Behnken design (BBD) based on the chosen key factors derived from the FFD. We assigned codes to these factors as mentioned previously. The design incorporated three levels (-1, 0, and 1, corresponding to the lowest, median, and highest values, respectively) and resulted in 17 experimental conditions, including five center points (representing the median values of all factors). These center points were essential for assessing the adequacy of the fitted model (Table 2). In total, we conducted 17 experiments, and the predictive model derived from response surface analysis, along with the generated three-dimensional response surfaces, vividly illustrated the influence of individual factors and their interactions on the extraction efficiency (Fig 4 and S2 Table in S1 File). Significantly, enhancing the volumetric ratio of MTBE in the extraction solvent resulted in improved extraction efficiency. Simultaneously, the volume of the extraction solvent and extraction duration demonstrated an efficiency trend, peaking at specific levels (500 μL and 25 minutes, respectively), beyond which efficiency declined. Nevertheless, to achieve maximum efficiency while minimizing extraction duration, we selected the likely optimal extraction conditions: using 500 μL of pure MTBE as the extraction solvent and an extraction duration of five minutes. We made this choice based on the predicted R value, which closely approached 100% (specifically, 103.19%). In a previous study, 1:7, the generic optimal volumetric ratio of the sample solution to the extraction solvent for LLE was suggested [35]. For this study, with a 20 μL plasma sample volume, we explored a range from 1:5 to 1:45, encompassing the previously suggested optimal ratio. Notably, RSM pinpointed a more effective ratio of 1:25, likely due to the fact that two-thirds of the critical factors in our method were interactions between factors. To validate the feasibility of the RSM-derived conditions, we conducted the R experiments five times under the likely optimal conditions, yielding an R value of 104.28 ± 4.08%. The consistent ratio of 101.06% affirms that the RSM effectively optimized the LLE conditions for the E/P steps of DPZ in rat plasma. We performed all experiments for DOE at a DPZ concentration of 125 ng/mL, and the data and results underwent analysis using Stat-Ease Design-Expert (Version 22, Minneapolis, MN, USA).

**Table 2:** Box-Behnken design matrix for three independent variables with coded values (in parentheses) and recovery (%): Volumetric ratio of methyl *tert*-butyl ether to ethyl acetate in the extraction solvent (%, A), volume of the extraction solvent (μL, B), and extraction duration (minutes, C).

Run	Independent variables			Response variable
	A (%)	B (μL)	C (minutes)	Recovery (%)
1	0 (-1)	100 (-1)	25 (0)	68.01
2	50 (0)	900 (1)	45 (1)	65.11
3	50 (0)	500 (0)	25 (0)	94.05
4	100 (1)	500 (0)	5 (-1)	102.54
5	0 (-1)	900 (1)	25 (0)	56.92
6	0 (-1)	500 (0)	5 (-1)	59.77
7	50 (0)	500 (0)	25 (0)	90.70
8	100 (1)	500 (0)	45 (1)	96.57
9	50 (0)	100 (-1)	5 (-1)	76.92
10	50 (0)	900 (1)	5 (-1)	72.12
11	50 (0)	500 (0)	25 (0)	91.29
12	100 (1)	900 (1)	25 (0)	91.90
13	50 (0)	100 (-1)	45 (1)	74.85
14	50 (0)	500 (0)	25 (0)	93.32
15	0 (-1)	500 (0)	45 (1)	65.54
16	100 (1)	100 (-1)	25 (0)	103.20
17	50 (0)	500 (0)	25 (0)	92.29

### Method validation

In the DPZ screening transition chromatogram, we observed a small peak-like bump (with a S/N of 1.17) at the retention time of DPZ (3.2 minutes) from the blank rat plasma results (Fig 2). Given that an S/N less than 3 suggests no detection, we excluded the possibility of DPZ contamination in blank rat plasma. Furthermore, the percentile ratio of the average bump area from blank plasma to the average DPZ peak area from the LLOQ QC sample was below 20% (12.39%). Consequently, we determined that there is no interference with DPZ, adhering to the guidelines. Regarding the IS, we did not observe any peak with the same retention time, affirming the selectivity of the current method (Fig 2). We verified the linearity of the method across the chosen concentration range (0.5, 1.5, 5, 50, 250, 500, 1,000 ng/mL) through six calibration curves. The back-calculated accuracy of calibrators fell within the range of 96.7% to 111.8% (with CV values less than 14.6%), satisfying the guideline criteria (the back-calculated accuracy of calibrators at LLOQ within 80–120%, and within 85–115% at all other concentrations, Table 3) [29]. Additionally, the r^2^ for the calibration curves was 0.9999 ± 0.013. For QC samples, SSSs were chosen over MMSs, as SSSs more closely resemble the actual final sample extract. Since a standard is added to the sample blank extract resulting from E/P in MMS, this approach is limited in reflecting the efficiency of analyte recovery by E/P. In contrast, SSS accurately reflects the analyte’s recovery by E/P, as the standard is added to the blank sample before E/P. From all QC samples, we determined the following accuracy and precision: intra-day accuracy ranged from 96.2% to 109.6%, inter-day accuracy from 96.0% to 107.1%, intra-day precision below 11.1%, and inter-day precision below 13.9% (Table 4). Consequently, all accuracy and precision outcomes aligned with the guideline criteria (The accuracy at each concentration level should be within ±15% of the nominal concentration, and within ±20% at the LLOQ. The precision of the measured concentrations at each level should not exceed 15%, except at the LLOQ where it should not exceed 20%.) [29]. Table 5 reveals that all ME results adhered to the guideline criteria: the mean ME ranged from 92.2% (at the HQC level) to 103.8% (at the LQC level) with CV values below 10.0% (at the LLOQ level) [29]. The ME results suggest that no chemical species would compromise the quantitation performance of the present method in the plasma extract. Thus, building calibration curves using simple standard solutions posed no issues; however, MMSs were employed to ensure greater accuracy. In the R experiments, all outcomes exhibited excellent R values near 100% (ranging from 98.5% to 106.8%) and consistent performance (with CV values below 4.60%) (Table 5). We confirmed the LLOQ for the present method as 0.5 ng/mL, the lowest concentration which satisfies the accuracy and precision criteria within the linear dynamic range. We evaluated the stability of DPZ across different conditions, and all findings aligned with the guideline criteria (S3 Table in S1 File). Following three freeze-thaw cycles at -80°C, DPZ in plasma demonstrated accuracy within the range of 99.8% to 114.1% against the respective nominal concentrations, with precision values falling below 7.3%. During benchtop stability testing in plasma at room temperature for four hours, DPZ exhibited accuracy ranging from 92.9% to 98.5% and precision within 10.5%. Over a four-week period, long-term stability testing of DPZ in plasma at -80°C revealed accuracy spanning from 88.5% to 96.7%, with precision falling within 7.0%. Upon reanalysis after 24 hours in an autosampler set at 4°C, the accuracy and precision of the extracted QC samples were within the ranges of 95.5% to 103.6% and below 5.9%, respectively. Finally, the stability assessment of working solutions at -20°C for one week yielded accuracy values ranging from 97.3% to 100.9%, with precision falling within 2.1%.

**Table 3:** Back-calculated accuracy (%) and its coefficient of variation (CV, %) of seven donepezil (DPZ) calibrators (n = 6). SD stands for standard deviation.

Nominal concentration of DPZ (ng/mL)	Calculated concentration of DPZ (mean ± SD, ng/mL)	Accuracy (%)	CV (%)
0.5	0.55 ± 0.08	111.79	14.56
1.5	1.48 ± 0.20	98.76	13.19
5	5.29 ± 0.57	105.86	9.89
50	48.33 ± 3.77	96.65	7.22
100	251.67 ± 3.03	100.67	1.20
500	499.99 ± 7.19	99.99	1.44
1,000	999.63 ± 3.31	99.96	0.33

**Table 4:** Accuracy and precision (coefficient of variation of accuracy) assessed from donepezil (DPZ) quality control samples. SD stands for standard deviation.

Types	Nominal concentration of DPZ (ng/mL)	Calculated concentration of DPZ (mean ± SD, ng/mL)	Accuracy (%)	Precision (%)
Intra-day (n = 6)	0.5	0.54 ± 0.06	109.58	11.09
	1.5	1.44 ± 0.09	96.19	6.30
	500	494.50 ± 13.83	98.90	2.80
	1,000	966.40 ± 16.33	96.64	1.69
Inter-day (n = 18, n = 6 for each day)	0.5	0.54 ± 0.07	107.13	13.90
	1.5	1.51 ± 0.13	100.71	8.93
	500	486 ± 23.55	97.20	4.85
	1,000	960.37 ± 25.31	96.04	2.64

**Table 5:** Matrix effect and recovery of donepezil (DPZ) in rat plasma (n = 6). SD and CV stand for standard deviation and coefficient of variation, respectively.

Nominal concentration of DPZ (ng/mL)	Recovery (mean ± SD, %)	Matrix effect	
		Mean ± SD (%)	CV (%)
0.5	102.99± 4.74	98.81 ± 9.90	10.01
1.5	98.46 ± 8.30	103.75 ± 6.31	6.08
500	104.71 ± 3.30	100.40 ± 3.64	3.63
1,000	106.79 ± 2.98	92.17 ± 2.44	2.64

### Comparison of the present method with recently reported methods

As part of evaluating the performance of our method, we compared it with nine recently reported methods using LC-MS/MS for determining DPZ in plasma [15–20,31,36,37]. Our primary objective in this study was to develop a novel method that combines high efficiency requiring less than 50 μL of plasma, high sensitivity surpassing the LLOQ of 1 ng/mL, and a wide dynamic range spanning at least three orders of magnitude. Thus, we conducted comparisons in these three aspects. For efficiency assessment, we compared the volume of sampling in each method, and the present method requires the smallest volume of plasma (20 μL) for sample preparation (Table 6). Since larger sampling volumes are typically intended to enhance sensitivity by enriching the analyte during the E/P steps, our method ranks seventh out of the ten methods when comparing LLOQs (Table 6). However, its LLOQ (0.5 ng/mL) comfortably meets the primary goal (less than 1 ng/mL) of the present study. To ensure broad applicability across various formulations (from oral to transdermal), a wide dynamic range spanning at least three orders of magnitude is crucial. Among the seven methods, only the present method and Bhateria et al.’s method achieved this dynamic range (Table 6) [15]. Therefore, our method’s excellent performance was confirmed through comparisons with the nine recently reported methods. It stands as the first analytical method for DPZ with three key qualities: high efficiency requiring less than 50 μL of plasma, high sensitivity surpassing the LLOQ of 1 ng/mL, and a wide dynamic range spanning at least three orders of magnitude. The fine optimization of E/P steps considering factor interactions using DOE likely played a significant role in achieving these results.

**Table 6:** Comparisons of the present method with recently reported methods using LC-MS/MS to determine donepezil in plasma. LLOQ, LLE, SLE, SPE, PPT stand for the lower limit of quantitation, liquid-liquid extraction, supported liquid extraction, solid phase extraction, and protein precipitation, respectively.

Reference	The volume of sampling (μL)	LLOQ (ng/mL)	Dynamic range (ng/mL) / the order of magnitude	Remarks
Park et al., 2008 [36]	200	0.1	0.1–50 / 2	Human/LLE
Iordachescu et al., 2012 [31]	200	0.05	0.05–25 / 2	Human/SLE
Katakam et al., 2013 [37]	300	0.05	0.05–25 / 2	Human/LLE
Bhateria et al., 2015 [15]	50	0.2	0.2–400 / 3	Rat/LLE
Mano et al.,2016 [16]	100	0.5	0.5–100 / 2	Human/SPE
Jeong et al.,2018 [17]	200	0.1	0.1–50 / 2	Human/LLE
Huang et al.,2022 [18]	200	0.2	0.2–50 / 2	Human/PPT
Yoon et al.,2023 [19]	30	1	1–200 / 2	Rat/PPT
Choi et al.,2023 [20]	30	2	2–500 / 2	Rat/PPT
The present method	20	0.5	0.5–1,000 / 3	Rat/LLE

### Applicability evaluation of the present method to a long-term pharmacokinetic study on DPZ formulations

**Fig 5:**
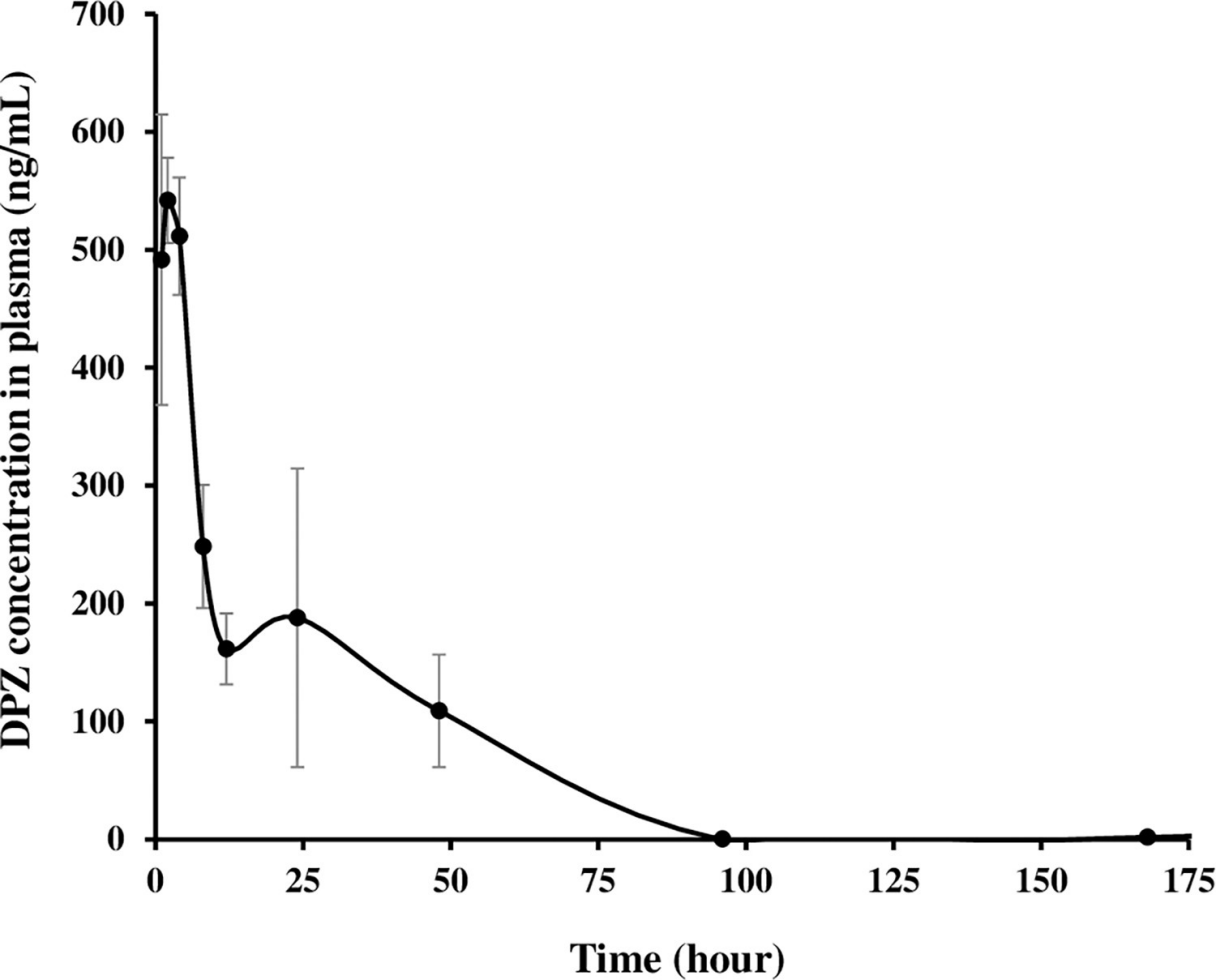
Plasma concentration-time profile of donepezil (DPZ) following intramuscular injection of drug suspension in normal rats (24.5 mg/kg as DPZ). Data represent mean ± standard deviation (n = 5).

The plasma concentration-time profile of DPZ obtained via the present method after IM injection is depicted in Fig 5. Additionally, the relevant pharmacokinetic parameters such as AUC, C_max_, T_max_, and elimination T_1/2_ calculated from the pharmacokinetic profiles are represented in Table 7. After IM injection, the level of DPZ in plasma increased and reached C_max_ (599.1 ng/mL) after 2.2 hour (T_max_). Subsequently, the drug concentration in plasma gradually decreased over 7 days with the extended elimination T_1/2_ of 23.1 hours. The AUC_(0–7 days)_ and AUC_inf_ values of DPZ, indicators of the extent of drug absorption, were determined to 12,436.7 and 14,336.2 ng·hr/mL, respectively. The plasma concentration of DPZ after 7 days of post-dosing was determined to be 1.8 ng/mL. Since all quantitation values (599.1 ng/mL-1.8 ng/mL) monitored over the long-term period (7 days) fell within the dynamic range of the present method (0.5–1,000 ng/mL), its applicability to actual pharmacokinetic studies on DPZ formulations was successfully confirmed. Consequently, the present method is actively being used for *in vivo* evaluations of several new formulations of DPZ in our research team.

**Table 7:** Pharmacokinetic parameters of donepezil (DPZ) following intramuscular injection of drug suspension in normal rats (24.5 mg/kg as DPZ). SD stands for standard deviation.

Parameters	Mean ± SD (n = 5)
C_max (_ng/mL)	599.1 ± 50.6
T_max_ (hr)	2.2 ± 0.8
AUC_(0–7 days)_ (ng∙hr/mL)	12,436.7 ± 2,318.1
AUC_inf_ (ng∙hr/mL)	14,336.2 ± 3,739.4
T_1/2_ (hr)	23.1 ± 23.7

## Conclusions

In this study, we propose an efficient, sensitive, wide-dynamic, and cost-effective method for determining DPZ in rat plasma. This method utilizes LLE followed by LC-MS/MS to facilitate *in vivo* evaluation of new DPZ formulations. Notably, the method requires only 20 μL of rat plasma and employs icopezil as the IS, which is both cost-effective and chemically similar to DPZ. The LLE conditions were meticulously optimized, considering factor interactions using DOE. By extracting DPZ from the sample using 500 μL of pure MTBE over a five-minute period, we achieved a fast and simple E/P. The dynamic range of the method spans from 0.5 ng/mL to 1,000 ng/mL, demonstrating high sensitivity and suitability for pharmacokinetic studies on various DPZ formulations. We rigorously validated the developed method in accordance with FDA guidelines, assessing selectivity, linearity, accuracy, precision, ME, R, LLOQ, and stability. Finally, we successfully applied the validated method to the long-term pharmacokinetic evaluation of a DPZ formulation. We anticipate that this approach will significantly contribute to the development of new DPZ formulations, benefiting individuals suffering from AD.

## Supporting information

S1 File Supplementary tables and figures on fractional factorial design, analysis of variance of the response surface analysis-derived predictive model, stability of donepezil, and optimization of liquid chromatography conditions. (DOCX)
