# Supplementary material for: Optimized method development and validation for determining donepezil in rat plasma: A liquid-liquid extraction, LC-MS/MS, and design of experiments approach
Source: PLoS One. 2024 Sep 6;19(9):e0309802. doi: 10.1371/journal.pone.0309802 (PMC11379286; doi:10.1371/journal.pone.0309802)

Supporting information for *PLoS One*

Optimized method development and validation for determining donepezil in rat plasma: A liquid-liquid extraction, LC-MS/MS, and design of experiments approach

Ye Bin Shin^1¶^, Jin Hwan Kim^1¶^, Min Kyeong Kwon^1^, Jin Hyuk Myung^1^, Dong Geon Lee^1^, Sung Giu Jin^2^, Myung Joo Kang^1*^, Yong Seok Choi^1*^

^1^College of Pharmacy, Dankook University, Cheonan, Chungnam 31116, South Korea

^2^Department of Pharmaceutical Engineering, Dankook University, Cheonan, Chungnam 31116, South Korea

^*^Corresponding authors

E-mail: kangmj@dankook.ac.kr (MJK) and analysc@dankook.ac.kr (YSC)

^¶^These authors contributed equally to this work.

Short title: DOE-optimized donepezil determination in rat plasma using LLE and LC-MS/MS

| **Run** | **Independent variables** | | | | **Response variable** |
| --- | --- | --- | --- | --- | --- |
|  | **A (%)** | **B (**$\boldsymbol{\mu L}$**)** | **C (minutes)** | **D (℃)** | **Recovery (%)** |
| 1 | 0 (-1) | 900 (1) | 45 (1) | 0 (-1) | 58.57 |
| 2 | 0 (-1) | 100 (-1) | 5 (-1) | 0 (-1) | 70.24 |
| 3 | 100 (1) | 100 (-1) | 5 (-1) | 40 (1) | 52.48 |
| 4 | 0 (-1) | 900 (1) | 45 (1) | 40 (1) | 45.76 |
| 5 | 0 (-1) | 100 (-1) | 45 (1) | 0 (-1) | 62.86 |
| 6 | 100 (1) | 900 (1) | 5 (-1) | 0 (-1) | 61.71 |
| 7 | 100 (1) | 900 (1) | 45 (1) | 0 (-1) | 58.16 |
| 8 | 100 (1) | 100 (-1) | 45 (1) | 40 (1) | 48.08 |
| 9 | 100 (1) | 100 (-1) | 45 (1) | 0 (-1) | 48.35 |
| 10 | 100 (1) | 900 (1) | 5 (-1) | 40 (1) | 50.99 |
| 11 | 0 (-1) | 900 (1) | 5 (-1) | 40 (1) | 22.92 |
| 12 | 0 (-1) | 100 (-1) | 5 (-1) | 40 (1) | 69.73 |

S1 Table. Fractional factorial design matrix for four independent variables with coded values (in parentheses) and recovery (%): volumetric ratio of methyl *tert*-butyl ether to ethyl acetate in the extraction solvent (%, A), volume of the extraction solvent (μL, B), extraction duration (minutes, C), and extraction temperature (°C, D)

| **Source** | **Sum of squares** | **Degrees of freedom** | **Mean square** | **F value** | **P-value** |
| --- | --- | --- | --- | --- | --- |
| **Model** | 3745.07 | 9 | 416.12 | 108.19 | < 0.0001 |
| A | 2590.54 | 1 | 2590.54 | 673.55 | < 0.0001 |
| B | 170.55 | 1 | 170.55 | 44.34 | 0.0003 |
| C | 10.75 | 1 | 10.75 | 2.8 | 0.1384 |
| AB | 0.0121 | 1 | 0.0121 | 0.0031 | 0.9569 |
| AC | 34.49 | 1 | 34.49 | 8.97 | 0.0201 |
| BC | 6.11 | 1 | 6.11 | 1.59 | 0.2478 |
| A² | 12.68 | 1 | 12.68 | 3.3 | 0.1123 |
| B² | 471.79 | 1 | 471.79 | 122.67 | < 0.0001 |
| C² | 379.38 | 1 | 379.38 | 98.64 | < 0.0001 |
| **Residual** | 26.92 | 7 | 3.85 |  |  |
| Lack of fit | 19.22 | 3 | 6.41 | 3.33 | 0.1378 |
| Pure error | 7.7 | 4 | 1.92 |  |  |
| **Cor total** | 3771.99 | 16 |  |  |  |

S2 Table. Results from analysis of variance (ANOVA) of the response surface analysis-derived predictive model (confidence level at 95%, p<0.05): volumetric ratio of methyl *tert*-butyl ether to ethyl acetate in the extraction solvent (%, A), volume of the extraction solvent (μL, B), and extraction duration (minutes, C)

| **Types of stability** | **Nominal DPZ concentration**  **(ng/mL)** | **Calculated DPZ concentration (ng/mL)** | **Stability (%)** | **CV**  **(%)** |
| --- | --- | --- | --- | --- |
| Plasma, freeze–thaw  (3 cycles, -80℃) | 1.5 | 1.71 ± 0.13 | 114.09 | 7.30 |
|  | 1000 | 997.71± 21.04 | 99.77 | 2.11 |
| Plasma, benchtop  (24 hours, 20℃) | 1.5 | 1.39 ± 0.15 | 92.88 | 10.50 |
|  | 1000 | 984.68 ± 38.04 | 98.47 | 3.86 |
| Plasma, long-term  (4 weeks, -80℃) | 1.5 | 1.33 ± 0.09 | 88.52 | 6.98 |
|  | 1000 | 966.83 ± 26.06 | 96.68 | 2.70 |
| QC samples  (24 hours, 4℃) | 1.5 | 1.55 ± 0.09 | 103.62 | 5.94 |
|  | 1000 | 954.58 ± 19.41 | 95.46 | 2.03 |
| Working solutions  (7 days, -20℃) | 1.5 | 1.45 ± 0.03 | 97.26 | 2.06 |
|  | 1000 | 1008.7 ± 1.58 | 100.87 | 0.16 |

S3 Table. Results from the evaluation of donepezil stability (n=6)

S1 Fig. Chromatograms comparing four mobile phase combinations with orgnic and acid modifiers (water and acetonitrile (A), water and methanol (B), 0.1% (v/v) formic acid in water and 0.1% formic acid (v/v) in acetonitrile (C), and 0.1% (v/v) acetic acid in water and 0.1% acetic acid (v/v) in acetonitrile (D)) for the analysis of a donepezil standard solution (10 ppb) through a Luna Omega Polar C18 column under gradient mobile phase conditions


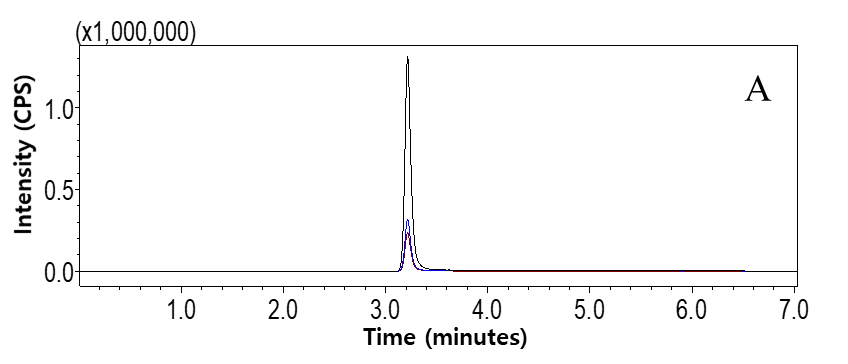


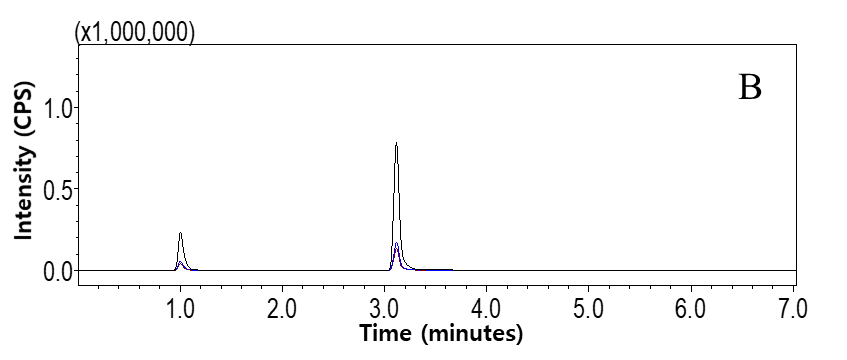


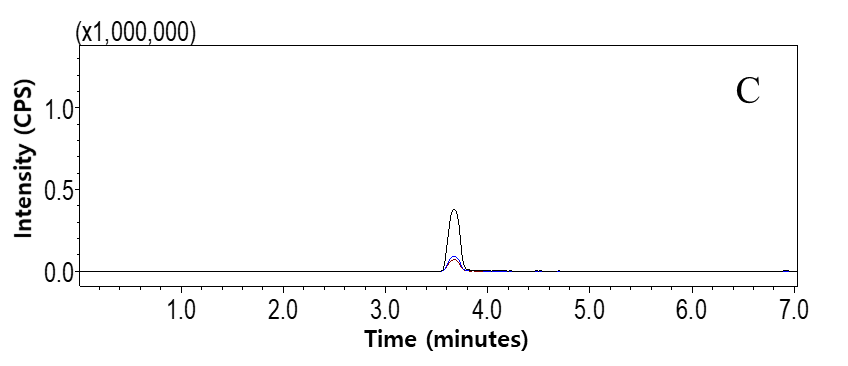


S2 Fig. Chromatograms comparing four mobile phase combinations with organic and acid modifiers: water and acetonitrile (A), water and methanol (B), 0.1% (v/v) formic acid in water and 0.1% formic acid (v/v) in acetonitrile (C), and 0.1% (v/v) acetic acid in water and 0.1% acetic acid (v/v) in acetonitrile (D), for the analysis of a donepezil standard solution (10 ppb) using a Luna Omega Polar C18 column under gradient mobile phase conditions


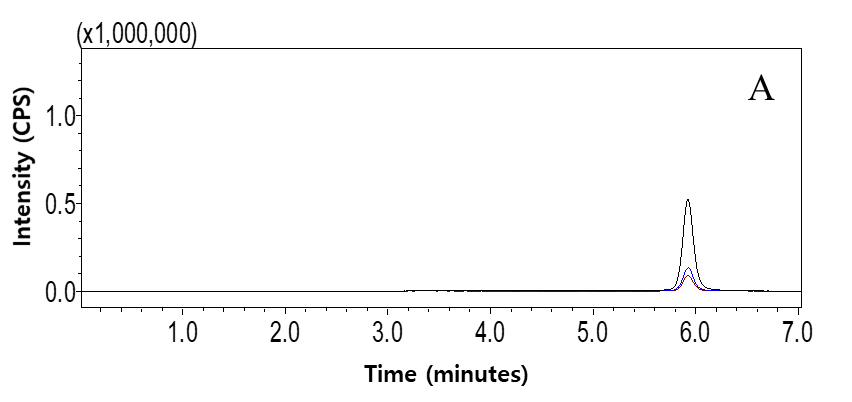


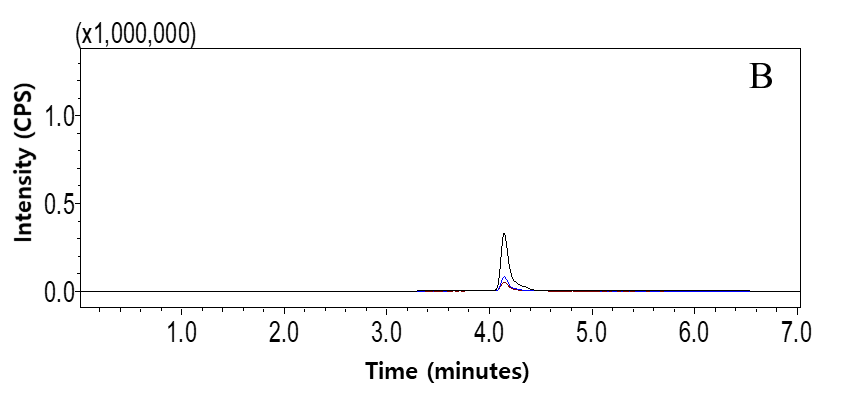


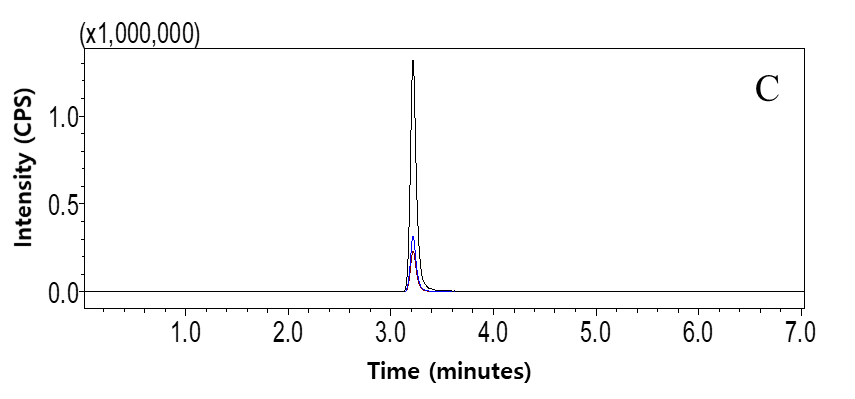


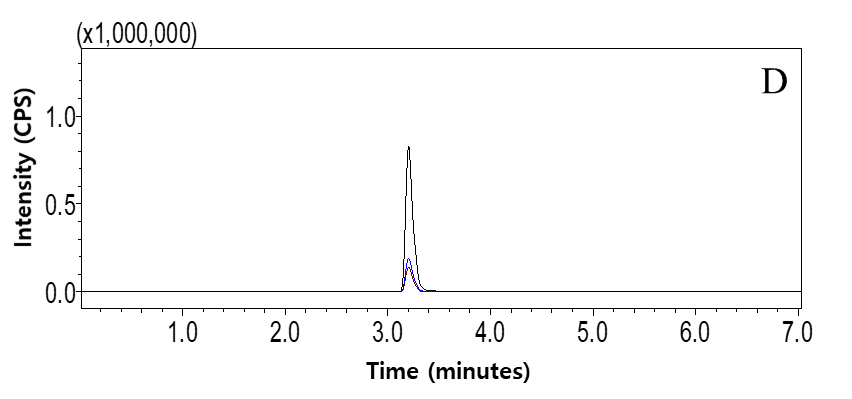

Supplement: S1 File — (DOCX) [file pone.0309802.s001.docx]
